# Automated Speech Intelligibility Assessment Using AI-Based Transcription in Children with Cochlear Implants, Hearing Aids, and Normal Hearing

**DOI:** 10.3390/jcm14155280

**Published:** 2025-07-25

**Authors:** Vicky W. Zhang, Arun Sebastian, Jessica J. M. Monaghan

**Affiliations:** 1 National Acoustic Laboratories, Sydney, NSW 2109, Australia; 2 Department of Linguistics, Macquarie University, Sydney, NSW 2109, Australia

**Keywords:** speech intelligibility, artificial intelligence, speech production, hearing loss, cochlear implants, hearing aids, children, natural language processing

## Abstract

**Background/Objectives**: Speech intelligibility (SI) is a key indicator of spoken language development, especially for children with hearing loss, as it directly impacts communication and social engagement. However, due to logistical and methodological challenges, SI assessment is often underutilised in clinical practice. This study aimed to evaluate the accuracy and consistency of an artificial intelligence (AI)-based transcription model in assessing SI in young children with cochlear implants (CI), hearing aids (HA), or normal hearing (NH), in comparison to naïve human listeners. **Methods**: A total of 580 speech samples from 58 five-year-old children were transcribed by three naïve listeners and the AI model. Word-level transcription accuracy was evaluated using Bland–Altman plots, intraclass correlation coefficients (ICCs), and word error rate (WER) metrics. Performance was compared across the CI, HA, and NH groups. **Results**: The AI model demonstrated high consistency with naïve listeners across all groups. Bland–Altman analyses revealed minimal bias, with fewer than 6% of sentences falling outside the 95% limits of agreement. ICC values exceeded 0.9 in all groups, with particularly strong agreement in the NH and CI groups (ICCs > 0.95). WER results further confirmed this alignment and indicated that children with CIs showed better SI performance than those using HAs. **Conclusions**: The AI-based method offers a reliable and objective solution for SI assessment in young children. Its agreement with human performance supports its integration into clinical and home environments for early intervention and ongoing monitoring of speech development in children with hearing loss.

## 1. Introduction

Speech intelligibility (SI) reflects the degree to which a speaker’s intended message is understood by listeners [1]. This is a critical skill for children to develop to engage in effective communication and participate fully in social interactions [2]. SI development depends on multiple factors, including speech perception, cognitive processing, linguistic knowledge, and articulation skills [3,4,5]. As an indicator of expressive language development, the assessment and improvement of SI are essential for early language development.

Typically developing children usually acquire SI gradually during early childhood. By the age of four, their speech is nearly 100% intelligible when rated by parents, with consistent results reported across studies using naïve listeners [6,7,8]. In contrast, children with hearing loss (HL), especially those with severe or profound HL, often face significant challenges in developing intelligible speech [9,10,11]. Studies have shown that speech produced by children with severe to profound HL is, on average, only 19–20% intelligible to naïve listeners, with wide variability reported based on the severity of hearing loss and the use of interventions such as hearing aids (HAs) or cochlear implants (CIs) [3,12,13].

The consequences of lower speech intelligibility may extend beyond communication difficulties for children with HL. Extensive research has shown that poor SI can affect how children with HL are perceived by their peers and teachers, hence negatively influencing their other areas of development, such as social functioning and overall psychosocial well-being [14,15,16,17,18,19]. Therefore, assessing and tracking SI development from early childhood through adolescence is essential for guiding effective interventions and supporting broader developmental progress [20].

Speech intelligibility measurement has been widely recognised as a useful method for assessing oral communication competence. However, considerable debate remains regarding the optimal approach for measuring it [21]. Factors such as the age and speech production stage of the child, the nature of the spoken material (e.g., isolated words or connected speech), and listener characteristics (e.g., familiarity with the speaker, listener’s hearing level, speech perception skills, and acoustic listening conditions) can all influence SI outcomes [22,23].

Current methods typically fall into two categories: transcription-based and scale-based procedures [20]. The transcription method requires listeners to write down the words or syllables they heard, with accuracy calculated based on the percentage of correctly transcribed items compared to the original speech stimulus [23,24]. While this approach provides detailed word-level analysis, it is time-consuming and normally requires multiple listeners for sentence transcription to ensure reliable scoring [24]. In the scale-based approach, listeners give an overall impression on a rating scale of how well the speech is understood. This method (e.g., a commonly used tool—the Speech Intelligibility Scale (SIR) is more time-efficient and has been widely adopted for assessing SI outcomes in children using HAs or CIs [25]. However, it provides less detailed information compared to transcription, and it may not be sensitive in distinguishing between moderate intelligibility levels, such as 40% versus 60% intelligible [26].

Regardless of the method used, SI judgments can be influenced by the familiarity and experience level of the listeners. If the goal is to reflect unbiased insights into children’s functional communication ability in daily life, researchers have consistently emphasised that the ideal evaluators should be inexperienced listeners with normal hearing and little or no exposure to the speaker’s speech [3,11,20,22,27,28,29,30,31]. However, recruiting panels of inexperienced listeners presents a practical barrier for widespread clinical implementation.

Artificial intelligence (AI) has proven transformative across various fields, with speech-to-text (STT) applications enabling advancements in voice recognition, transcription, and accessibility technologies. AI models like Whisper Large V2 have shown exceptional ability to perform automated transcription tasks with high accuracy and consistency. Unlike traditional methods, AI tools can provide efficient and objective evaluations, presenting a promising opportunity to improve SI assessments in clinical settings.

In recent research, AI-based STT technologies have been successfully applied to assess SI and related outcomes across various populations. For instance, one study used Automatic Speech Recognition (ASR) systems to evaluate speech impairment severity in oral cancer patients, focusing on intelligibility and voice quality through listener ratings [32]. Another explored a deep neural network (DNN)-based model for predicting SI in noisy environments, aimed at improving audiometry and hearing aid optimisation [33]. A separate study applied the Kaldi ASR toolkit to automate the digits-in-noise test, creating an accessible, efficient hearing assessment tool for clinical and remote settings [34]. Additionally, researchers have examined the feasibility of an automated system for assessing SI in individuals with aphasia, focusing on clarity, fluidity, and prosody through DNN models and feature engineering, showing potential for providing accurate, accessible feedback in aphasia therapy [35]. One study examined the effectiveness of AI-based synthesised speech as an alternative to human-recorded speech in speech-in-noise perception research. Results showed that both younger and older adults experience similar masking-release benefits with AI and human speech, with older adults perceiving AI speech as particularly natural and human-like, suggesting its potential value for research focused on ageing and speech perception [36]. Furthermore, recent work has shown that Natural Language Processing (NLP) models, such as OpenAI’s Ada2, as accurate alternatives to human scoring in SI evaluations, particularly under noisy conditions [37]. Collectively, these studies highlight the expanding role of STT technologies in enhancing clinical precision and accessibility in hearing and speech assessments. This development aligns with recent work exploring AI-based predictive systems in paediatric care settings, such as the use of multilayer perceptron models for early self-care prediction in children with disabilities [38].

This study aimed to address the challenges in SI assessment by leveraging AI technology. Specifically, the two primary objectives were to apply an AI-based transcription method for the automatic assessment of SI performance in 5-year-old children with CIs, HAs and normal hearing (NH); and to compare the word-level accuracy and consistency of AI-based transcriptions with those of naïve listeners for children across hearing groups.

## 2. Materials and Methods

### 2.1. Speech Samples

The speech materials were sentences from the Beginners Intelligibility Test (BIT) [10], a widely used tool for evaluating speech production in children with HL [10,23,39]. The BIT consists of four lists, each containing ten simple sentences, ranging from two to six words (three to eight syllables) and including 37 to 40 familiar words for young children, such as “The boy is under the table” and “That is a big bed”.

Speech samples were collected from 58 five-year-old children (male: 23, female: 35; mean age: 61.4 months, SD: 1.3), as part of a previous study [40], which was approved by the Australian Hearing Human Research Ethics Committee on 13 March 2012 (approved number: AHHREC2012-7). Consent from guardians of children was obtained as part of the original ethics approval. Each child was randomly assigned one of the four BIT lists. During test administration in a quiet room, a research speech pathologist read each sentence aloud using a picture cue to provide context of the target sentence, and the child was instructed to repeat the sentence. Audio recordings were captured using a Zoom H4N digital recorder with AKG C555L headset microphone at a 44.1 kHz sampling rate. Each child’s 10-sentence recording was further segmented into 10 individual files, with one second of silence before and after each sentence. All recordings were then normalised for root-mean-square (RMS) level using Adobe Audition software (v4.0), resulting in a total of 580 individual audio recordings: 100 from children with CIs, 240 from children using HAs, and 240 from children with NH. Table 1 shows the demographic and clinical characteristics of the 58 children. Among these children, 24 had NH, while 34 had bilateral HL with average air conduction hearing threshold across four frequencies (4FAHL) of 0.5, 1, 2, and 4 kHz in the better ear at 68.8 ± 31.1 dB HL. In the HL group, 10 had bilateral CIs and 24 children used bilateral HAs, with all devices fitted before age three. All children with HL were diagnosed through universal newborn hearing screening and had been consistently using their hearing devices since initial fitting. At the time of speech sample collection, all participants were active users of their devices, and none experienced progressive hearing loss between birth and the 5-year assessment. All children were native English speakers with a monolingual background and had no speech issues or additional disabilities according to parental reports.

### 2.2. Speech Intelligibility Evaluation by Naïve Listeners

All RMS-normalised recordings were presented via Beyerdynamic DT770 Pro circumaural headphones using the MACarena experiment presentation software [44] (https://www.orl.uzh.ch/projects/speechtests/speechtests.html, accessed on 15 July 2025). The listeners were 64 adults who were native speakers of Australian English aged between 18 and 40 years old (mean: 20.6 years, SD: 3.4). All adult listeners had passed screening tests with normal hearing and reported no previous exposure to speech produced by children with HL.

Each child’s 10-sentence BIT list was listened to and transcribed by at least three adult naïve listeners independently. Each listener was randomly assigned to transcribe four different BIT lists, each from a different child, with randomised sentence order within each list. After hearing each sentence, listeners provided word-by-word transcription based on what they believed the child had said. If necessary, they were allowed to replay the sentence once. For words or sentences completely unintelligible, they were instructed to either make their best guess or mark it with an ‘X’. Listeners did not hear the same list more than once nor did they listen to a list spoken by the same child more than once. Each listener was assigned recordings from at least one child with NH and one child with HL within the four lists. When more than three transcriptions were available for a child, a random subset of three was used for analysis. The data collection was conducted as part of a previous student project in collaboration with Macquarie University. Ethical approval for this part was granted by the Macquarie University Human Research Ethics Committee on 10 July 2018. Participant consent for the original studies was obtained under the approved ethics.

### 2.3. Selection of AI-Based Speech-to-Text Model

Speech transcription for paediatric populations presents unique challenges compared to adult speech. While state-of-the-art (SOTA) STT models demonstrate impressive performance on adult speech corpora, their direct application to children’s speech often remains limited. This discrepancy arises from several factors. Firstly, STT models are predominantly trained on large-scale datasets comprised almost entirely of adult speech, which differs acoustically from children’s speech due to anatomical differences such as shorter vocal tract length, higher fundamental frequency, and more variable articulation. As a result, models trained on adult speech may fail to generalise to the acoustic patterns characteristic of young children. Children’s speech corpora are significantly smaller than the extensive datasets used to train adult-focused ASR systems, limiting opportunities for robust model training and domain adaptation. Secondly, children’s speech introduces linguistic complexities, such as ungrammatical or poorly constructed sentences, disfluencies (e.g., false starts, filler words, and pauses), and speech sound errors, all of which pose transcription challenges [45,46].

**Figure 1:**
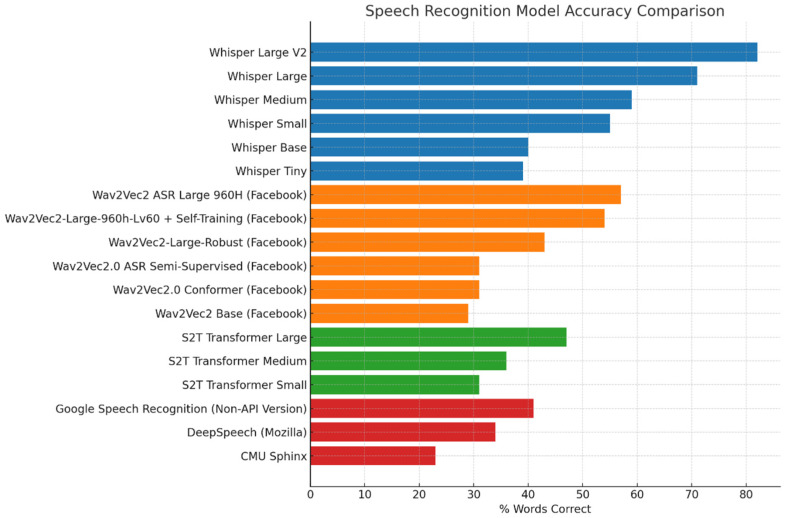
Transcription performance of evaluated Speech-to-Text (STT) Models on NH children’s speech recordings.

To identify a suitable AI-based model for evaluating intelligibility in children’s speech, we conducted a comparative analysis of a range of SOTA STT models. Due to privacy and ethical considerations associated with the speech recordings used in this study, only offline deployable models were considered, excluding any transcription systems that required internet-based data processing. Within this constraint, we selected five STT models based on their technical strengths, availability and relevance to clinical or paediatric speech contexts: Whisper [47], Wav2Vec 2.0 [48], S2T Transformer [49], DeepSpeech [50], and an offline implementation of Google Speech Recognition (non-API) model (Figure 1). Whisper, developed by OpenAI, is known for its robustness to acoustic variability, including background noise, accents, and disfluent speech. These characteristics make it particularly well-suited for handling the unpredictable pronunciation patterns and articulation variability commonly observed in children’s speech. Wav2Vec, with its self-supervised learning framework, excels in scenarios with limited annotated data, making it particularly advantageous for paediatric speech transcription applications where large-scale labelled datasets are scarce. The S2T Transformer models, based on a sequence-to-sequence architecture, are designed for direct speech translation and transcription tasks. They demonstrate efficient decoding and strong performance in multilingual and low-resource settings, which would be valuable for handling children’s speech variability. These models benefit from extensive pretraining on diverse speech corpora, resulting in high baseline accuracy across a range of accents and conditions. Finally, DeepSpeech, while based on an earlier recurrent neural network (RNN) architecture, remains a lightweight and adaptable open-source model useful as a flexible baseline for comparison. Together, these models represent a diverse cross-section of contemporary speech recognition approaches, allowing us to assess the overall feasibility of automated transcription for children’s speech using existing, offline-deployable tools. CMU Sphinx [51] was included to represent a non-neural, rule-based ASR approach and to provide a performance contrast with modern deep learning-based models.

The SOTA models were accessed via the Hugging Face Transformers library and implemented using a Python-based framework (v3.10). Audio inputs were pre-processed and handled using the torchaudio library, and model inference was performed using PyTorch (v1.13). For CMU Sphinx, transcription was performed using the SpeechRecognition Python package, which provides a wrapper for the Sphinx engine as a lightweight, offline baseline comparator. Transcriptions were generated for speech recordings produced by children with NH, and the performance was quantified as the percentage of correctly transcribed words relative to the reference BIT lists. Specifically, a percentage score was calculated for each child by dividing the total number of correctly transcribed words across all 10 sentences by the total number of words in the BIT list. The final transcription performance for each model was then computed by averaging these percentage scores across all tested children’s recording (Figure 1). Among the models tested, the Whisper Large V2 achieved the highest overall accuracy. Additional comparisons using speech samples from children with HL provided consistent results, with Whisper Large V2 continuing to outperform all other tested models in transcription accuracy. These findings support its suitability for application across both NH and HI populations. Therefore, it was selected for all subsequent transcription and analyses in this study.

### 2.4. Data Scoring on the Transcriptions

All target words in the BIT list were weighted and scored equally [23,30,52]. For each sentence, the transcribed words were compared to the original BIT reference lists. This scoring method was applied identically across both naïve listeners and the AI model to ensure comparability. The transcriptions by naïve listeners were double-checked for correct children’s and naïve listeners’ IDs, correct audio recording file, list number, and sentence number by two researchers. For each sentence produced by each child, the word-by-word transcription provided by each naive listener was verified independently by two researchers to ensure consistency, with reference to the BIT lists. The AI-based transcriptions were automatically processed using the offline Whisper Large v2 model and scored using the same method as the transcriptions by adult naïve listeners. Therefore, each child’s sentence list has a 4th transcription result generated by the AI-based method. Here is an example from an NH child’s speech sample. The original sentence was “*The boy walked to the table*”, and the three naïve listeners transcribed it as follows: “*The boy walked to the table*” (Listener 1), “*The boy walked in a table*” (Listener 2), and “*The boy walked at a table*” (Listener 3). The transcription generated by the Whisper large v2 model was “*The boy walked to the table*”, which was fully correct and matched the reference as well as the listener 1. In contrast, the transcriptions by naïve listeners 2 and 3 each had four out of six words correctly matched to the reference sentence.

### 2.5. Data Analysis

The statistical analyses were performed using IBM SPSS Statistics for Windows v.29 [53].

To evaluate the agreement between transcriptions from naïve listeners and the AI-based method, Bland–Altman plots were constructed [54]. These plots visualised the agreement between paired transcriptions by plotting the differences against the mean of the two measures (i.e., the AI model relative to naïve listeners’ transcriptions), indicating mean biases or outliers based on the 95% limits of agreement (LoA).

To assess the overall consistency and reliability of transcriptions across different raters (three naïve listeners and the AI-based model), word-level Intraclass Correlation Coefficient (ICC) analyses were performed. These ICC analyses were conducted separately for children with CIs, HAs, and NH, to evaluate how well the AI-based method aligned with human performance across different hearing groups. Two-sided *p*-values < 0.05 were used to indicate statistical significance for ICC analysis to allow for the possibility of differences in either direction. No correction for multiple comparisons was applied, as the analyses were limited to the predefined comparisons. Although the ICC assumes normally distributed measurement differences, it is generally robust to moderate deviations from normality, particularly in larger samples such as the current study. ICC was therefore deemed appropriate for evaluating inter-rater agreement in this context [55].

The Word Error Rate (WER) metric, as a recognised benchmark, has been widely used to evaluate the reliability performance of automated STT models in diverse contexts (e.g., [56]). In this study, we also calculated the WER values for the AI model and naïve listeners across hearing groups to further quantitatively assess the transcription errors in terms of substitutions, deletions, and insertions relative to the total number of words in the reference transcription (Equation (1)):

(1) $$WER=\left(\frac{S+D+I}{N}\right)\times 100$$

where S = the number of substitutions; D = the number of deletions; I = the number of insertions; N = the total number of words in the reference text.

## 3. Results

### 3.1. Comparing Word-Level Transcription: AI vs. Naïve Listeners

**Figure 2:**
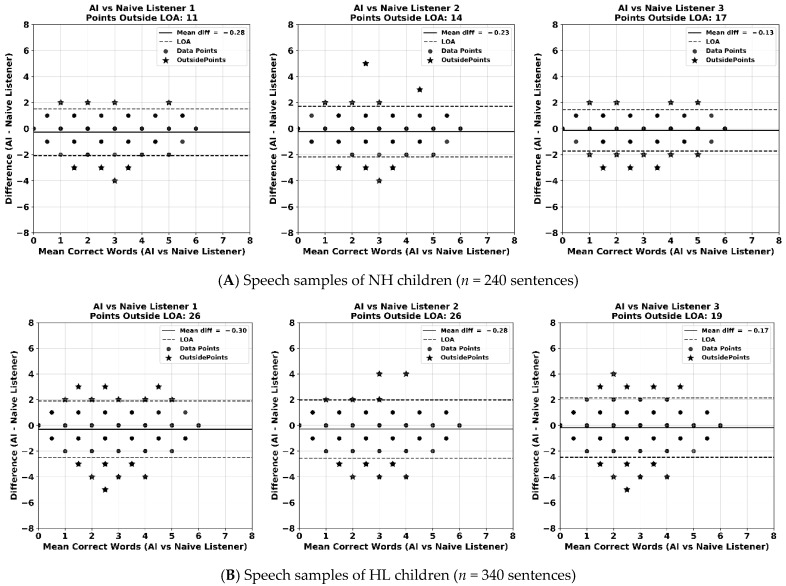
Bland–Altman plots of number of correct words between AI model vs. naïve listeners (difference plotted against the averaged number of correct words). The solid line represents the mean difference. The area within 2 dashed lines represents the upper and lower 95% limits of agreement (LOA). Panels (**A**,**B**) indicate the results from children with NH (*n* = 240 sentences) and those with HL (*n* = 340 sentences), respectively. The x-axis in each figure represents the average number of correct words between each naïve listener and the AI model, and the y-axis represents their difference score.

The Bland–Altman plots (Figure 2) show the comparison of word-level transcription agreement between the AI-based transcription model and individual naïve human listeners. The results are displayed separately for speech samples from children with NH (Figure 2A) and those with HL (Figure 2B). Each plot illustrates the average number of correct words between the AI model and a naïve listener against the difference score between the two methods, as well as the 95% limits of agreement (i.e., limits within which 95% of difference scores will lie).

Across all comparisons, the mean differences between the AI model and human listeners were close to zero, indicating minimal systematic bias in transcription accuracy. The plots also revealed a symmetrical distribution of data points above and below the zero-difference line, which suggests that neither transcription method consistently over- or under-performed relative to the other.

For children with NH, 95% LoA ranged from 4.6% to 7.1%, while for children with HL, the LoA were slightly wider, between 5.6% and 7.6%. For example, in the comparison between the AI model and naïve listener 1, only 4.6% sentences produced by NH children (11 out of 240) and 7.6% of sentences produced by HL children (26 out of 340) fell outside the 95% LoA. In comparison with naïve listener 3, 7.1% of NH sentences (17/240) and 5.6% of HL sentences (19/340) fell outside the 95% LoA. These patterns suggest that overall agreement between AI model and human listeners was strong, with slightly greater variability in children with HL, possibly reflecting differences in speech clarity and articulation across populations.

### 3.2. Word-Level Consistency Analysis Between the AI Model and Naive Listeners

The consistency of word-level transcription accuracy between the AI model and naïve listeners was assessed using the ICC, with the AI model treated as an additional rater alongside the three naïve listeners. To evaluate inter-human agreement independently, ICC values for the naïve listeners alone were also calculated. This distinction allows for clear comparison between human–human and human–AI consistency across hearing groups (Table 2).

In the NH group, inter-rater agreement was notably high. The ICC value among naïve listeners reached 0.95, with the AI model demonstrating comparable consistency, achieving an ICC of 0.96 when compared to human listeners.

For the HL group, which combines children with CIs and HAs, word-level transcription reliability remained high, though slightly lower than that observed in the NH group. The ICC among naïve listeners was 0.92, and the AI model maintained a closely aligned value of 0.93. Looking more closely at subgroups within the HL group, children with CIs showed performance that was directly comparable to NH peers. Both naïve listeners and the AI model achieved ICC values of 0.96, indicating a high degree of consistency in word-level transcription accuracy and suggesting relatively stable speech production in the CI group. In contrast, the HA subgroup showed the lowest but still acceptable levels of agreement. The ICC values for naïve listeners and the AI model were 0.90 and 0.91, respectively, which suggests a slightly greater variability in transcription accuracy.

### 3.3. Word Error Rate Consistency Among AI and Naïve Listener Transcriptions

**Figure 3:**
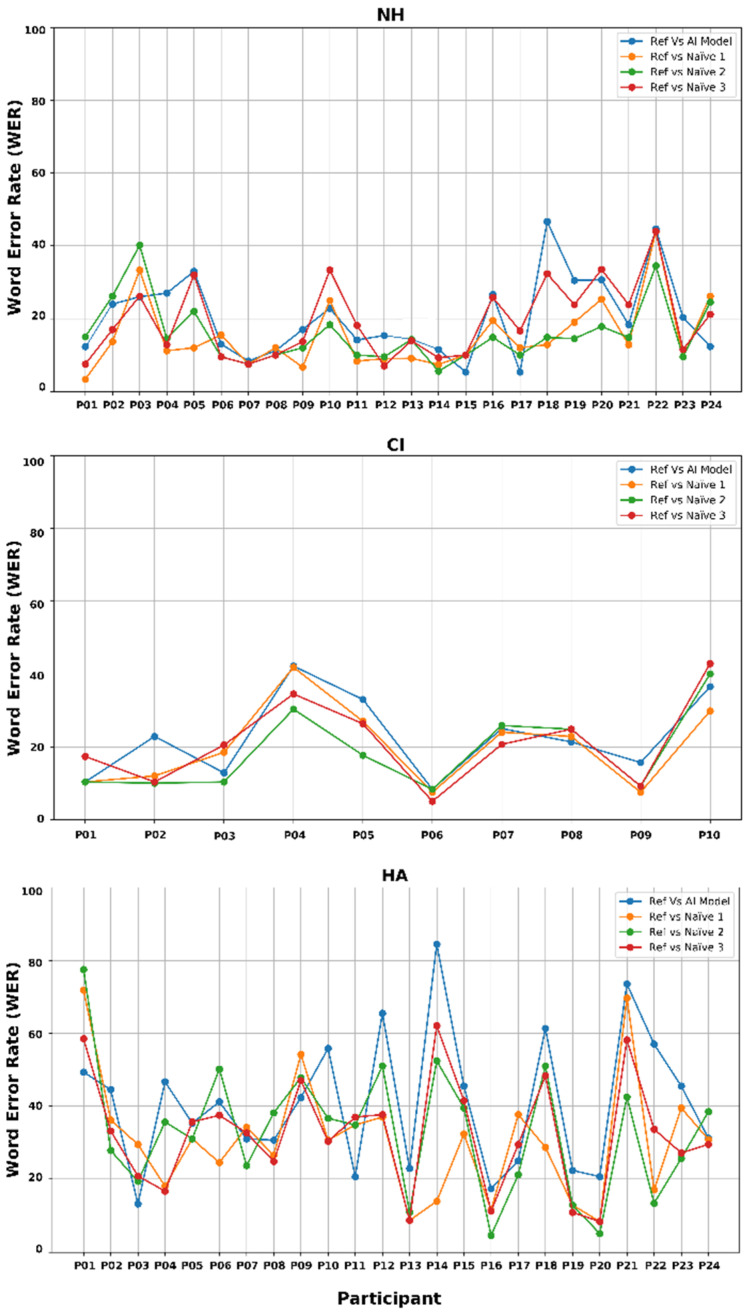
Average Word Error Rate (WER) per participant across transcription methods for children with NH, CIs and HAs.

To further evaluate transcription accuracy, word error rate was calculated by comparing each transcription generated by the AI model and individual naïve listeners, against the reference sentences on a word-by-word basis. This analysis aimed to assess the variability in word-level errors at both the participant level and transcription methods, across different hearing groups. Figure 3 illustrates the average WERs across all 10 sentences produced by each participant. WERs are shown for four transcription methods: the AI model compared to the reference sentence (labelled as “Ref vs. AI”), and each of the three naïve listeners compared to the reference (labelled as “Ref vs. Naïve 1, 2, or 3”). The results are displayed across three hearing groups (i.e., NH, CI, and HA) in separate subfigures. These visualisations allow for a clearer understanding of performance differences both within and between groups. Lower WER values indicate greater transcription accuracy.

In the NH group, WERs were low across all transcription methods, indicating high intelligibility of children’s speech. Among naïve listeners, listener 1 achieved the lowest average WER (15.2%), followed by listener 2 (15.9%) and listener 3 (19.2%). The AI model’s transcribing performance closely aligned with human listeners, with an average WER of 20.5%.

In the CI group, WER consistency was comparably high among transcription methods. The AI model achieved a WER of 22.8%, while naïve listener performances were 20.1% (listener 1), 18.7% (listener 2), and 21.2% (listener 3). This result suggests that, despite having severe to profound hearing loss, children with CIs produced speech that was as intelligible—and as consistently transcribed—as their NH peers. The AI model’s performance in transcribing speech from children with CIs was nearly equivalent to that of the human listeners, highlighting its effectiveness in handling speech from CI users.

By contrast, the HA group exhibited more variable and generally higher WERs across all transcription methods. Naïve listeners performed slightly better with WERs of 30.8% (listener 1), 32.8% (listener 2), and 32.3% (listener 3), compared with the AI model’s result of 40.7%. While no statistical tests were conducted to compare these values, the observed WER difference of approximately 8–10 percentage points may be clinically meaningful, particularly in populations with greater speech variability [21,57]. The higher WERs in this subgroup likely reflect greater variability in speech production among children with moderate–severe hearing loss and wearing hearing aids. This increased variability appears to challenge both human and AI transcription. Although the AI model produced higher WER than human listeners, the consistent trend across methods suggests it reliably tracks intelligibility patterns, even under more variable acoustic conditions.

## 4. Discussion

### 4.1. Importance of SI Evaluation for Children with HL

Speech intelligibility, as a critical benchmark for expressive speech-language development, reflects how well a child’s speech is understood by listeners and is essential for effective communication in social contexts. Despite its importance, SI assessments are often underutilised in clinical practice due to logistical and methodological challenges. Early identification of SI issues during early childhood, a sensitive period for speech and language development, could allow for timely interventions, guiding appropriate clinical management and family support strategies [20]. Thus, accurate and accessible methods for assessing SI are important for children with HL to monitor intervention progress and optimise language outcomes.

### 4.2. Summary and Interpretation of Current Findings

This study investigated the transcription accuracy and consistency of an AI-based model (Whisper Large V2), in comparison to naïve human listeners. The integration of multiple analyses approaches underscores its clinical relevance, with the Bland–Altman analysis providing word-level precision, ICCs offering an overall consistency across transcription methods in different hearing groups of children, and WERs capturing variations in word-level transcription errors. The findings indicate that the AI model performs consistently and comparably to human listeners in transcribing children’s speech.

The Bland–Altman analysis demonstrated strong agreement between the AI model and individual naïve listeners, with minimal bias and most sentences falling within the 95% LoA for both NH and HL groups. These findings suggest a high degree of transcription consistency in the word level across methods. The ICC analysis provided converging evidence, with values exceeding 0.90 in all groups—a threshold commonly interpreted as “excellent” reliability [55]. In addition, it was noted that the AI model demonstrated the highest consistency with human listeners in the NH and CI groups, achieving ICC values of 0.95 and 0.96, respectively, matching the performance of listeners. Although slightly lower agreement was observed in the HA group (ICC = 0.91), likely reflecting greater variability in speech intelligibility, these results underscore the robustness of the AI model across varied speech production characteristics and its close alignment with human transcription performance.

The WER analysis further supported these findings by quantifying transcription errors. In both the NH and CI groups, WERs for the AI model fell within the similar range as those of naïve human listeners, generally under 25%, a general indicative threshold widely accepted as adequate transcription quality [57]. Although transcription errors were more frequent in the HA group, the AI model performed consistently with the relative trends of human listeners, which suggests its potential for reliably evaluating speech intelligibility despite the challenges posed by less clear speech production.

A particularly promising finding was that both ICC and WER analyses showed that children with CIs achieved equivalent speech production outcomes to their NH peers, and better outcomes compared to their peers with severe hearing loss who use HAs. This reinforced findings in the literature that children fitted early with CIs may demonstrate good speech intelligibility and language skills that are comparable with those of typically hearing children [58,59,60,61]. These results also underscore the benefits of early CIs and consequence intervention in supporting speech intelligibility development in children with severe to profound HL.

Overall, these findings validate the selected AI model (Whisper Large V2) as a consistent and effective tool for word-level transcription of children’s speech. Its capability of achieving comparable performance with naïve human listeners, even in more degraded speech conditions, supports its use as a valuable tool for SI evaluation in clinic and home environments.

### 4.3. Strengths and Limitations

This study provides robust evidence for the potential of the selected AI model (i.e., Whisper Large V2) as an objective, effective, and reliable tool for the automated transcription of children’s speech recordings. The model’s consistently superior performance compared to other AI models across both NH and HI samples supports its utility as a robust STT tool for evaluating children’s speech intelligibility in different listening contexts (see Section 2.3). The use of three analysis metrics (Bland–Altman, ICC, and WER) adds methodological rigour and provides a nuanced understanding of AI-based model performance. Given the absence of AI models specifically trained on children’s language, the accuracy levels achieved by Whisper Large V2 offer valuable insights into the feasibility of using existing AI models for the automatic recognition and transcription of paediatric speech. Another strength of this study lies in its use of rigorous consistency analyses, comparing AI-generated transcriptions to those of naïve human listeners across multiple groups of children, including those with NH, and children who use CIs or HAs. The inclusion of a diverse cohort of children enhances the relevance of the findings for clinical applications.

Despite the strengths of this study, several limitations should be acknowledged. First, the dataset was limited to speech recordings from 5-year-old native English-speaking children without reported speech or developmental difficulties. While this controlled design improves internal validity, it restricts generalizability to other age groups, linguistic backgrounds, and children with developmental challenges. Second, the sample size of the CI subgroup was relatively small. Although consistent patterns were observed, the limited number may reduce statistical power for subgroup comparisons and constrain the generalisability of the results to broader CI populations. Future studies should aim to include larger and more diverse cohorts to further validate the robustness of these findings across a broader range of developmental profiles, device users, and linguistic contexts. Additionally, while Whisper Large V2 performed well without specific training on paediatric speech, fine-tuning the model with an age-appropriate cohort and optimising model parameters to better capture child-specific acoustic patterns [62] may further improve its performance. Finally, the current study focused on word-level transcription accuracy using the BIT materials. Future work could explore other types of speech materials (e.g., spontaneous speech) or phoneme-level analysis to expand its potential in clinical applications. Expanding evaluation to these aspects would better reflect real-world communication demands and further inform the development of automated SI tools for children with HL.

### 4.4. Future Clinical Application

The results of this study also point toward practical, scalable applications of AI for speech intelligibility assessment. In particular, the Whisper Large V2 model demonstrates the potential for integration into a telehealth platform or a user-friendly mobile apps, to support routine monitoring of children’s speech. Such tools could reduce clinician workload, enable more frequent tracking of speech development progress, and empower caregivers to participate actively in home-based interventions. To support wider deployment across clinical settings while preserving patient privacy, emerging technologies such as Federated Learning may facilitate future implementations by enabling scalable integration without sharing sensitive patient data [63].

Recent advancements by Monaghan et al. [64] have demonstrated that speech-based machine learning models, such as those using wav2vec 2.0 embeddings, can be used not only for assessing intelligibility but also for detecting hearing loss itself directly from children’s speech samples. This highlights a broader diagnostic potential for AI-based systems—enabling integrated approaches to both assess intelligibility and identify underlying hearing conditions, all from the same speech data [64].

By addressing current limitations and driving adoption through user-centre design, the AI-based SI tool has the potential to transform how speech outcomes are evaluated in children with hearing loss, extending benefits from clinic to home and ultimately improving long-term communication outcomes.

## 5. Conclusions

This study demonstrated that the AI-based transcription model (Whisper Large V2) is a reliable and objective tool for the automated assessment of speech intelligibility in young children. Its performance closely matched that of naïve listeners across hearing groups, with the highest consistency observed in children with NH and those using CIs. Notably, CI users demonstrated intelligibility levels comparable to their NH peers and higher than HA users, highlighting the importance of early amplification and intervention. The scalability and consistency of the AI model make it a promising tool for integration into clinical practice and home-based SI evaluations via telehealth platforms, which could further support early intervention and ongoing monitoring of speech development in children with hearing loss.

## Figures and Tables

**Table 1:** Demographic information of child talkers.

Characters	Cochlear Implant (CI) (*n* = 10)	Hearing Aid (HA) (*n* = 24)	Normal Hearing (NH) (*n* = 24)
Age at BIT assessment (months), Mean (SD)	61.4 (1.6)	61.4 (1.4)	61.5 (1.5)
Gender (Male), *n* (%)	3 (30.0%)	10 (41.7%)	10 (41.7%)
Degree of hearing loss at BIT assessment (4FA HL in better ear), Mean (SD)	109 (18.4)	52 (15.1)	na
Age at hearing aids fitting (months), Mean (SD)	5.3 (5.3)	5.2 (6.0)	na
Age at cochlear implantation (months), Mean (SD)	21.8 (16.5)	na	na
Nonverbal cognitive ability *, Mean (SD)	102.2 (14.7)	92 (13.5)	103.9 (15.8)
Language score *, Mean (SD)	104.7 (9.7)	110.2 (11.9)	104.6 (9.8)

Note *: Children’s nonverbal cognitive ability was evaluated by a research psychologist using the Wechsler Nonverbal Scale of Ability (WNV; [41]). Their expressive and receptive language skills were assessed by research speech pathologists using the Pre-school Language Scale, 4th edition (PLS-4; [42]). Both assessments were conducted within the 5-year follow-up interval of the LOCHI study [43].

**Table 2:** Intraclass Correlation Coefficients (ICCs) of word-level accuracy across hearing groups for naïve listeners and AI vs. human listeners’ comparisons.

Hearing Group	Comparison	ICC Value	95% Confidence Interval	*F (df1, df2)*	*p*-Value
NH group (*n* = 240 sentences)	Within naïve listeners only	0.95	[0.94, 0.96]	*F* (239, 478) = 20.8	<0.001
	AI model vs. naïve listeners	0.96	[0.95, 0.96]	*F* (239, 717) = 22.4	
HL group (CI and HA) (*n* = 340 sentences)	Within naïve listeners only	0.92	[0.90, 0.93]	*F* (339, 678) = 11.7	
	AI model vs. naïve listeners	0.93	[0.91, 0.94]	*F* (339, 1017) = 13.8	
CIs group only (*n* = 100 sentences)	Within naïve listeners only	0.96	[0.94, 0.97]	*F* (99, 198) = 20.8	
	AI model vs. naïve listeners	0.96	[0.94, 0.97]	*F* (99, 297) = 22.1	
HAs group only (*n* = 240 sentences)	Within naïve listeners only	0.90	[0.87, 0.92]	*F* (239, 478) = 9.6	
	AI model vs. naïve listeners	0.91	[0.89, 0.93]	*F* (239, 717) = 11.6	

Abbreviations: NH, normal hearing; HL, hearing loss; CIs, cochlear implant; HAs: hearing aids.

## Data Availability

Because the datasets are not available outside of the research team as per Hearing Australia Ethics approvals. Requests to access the datasets should be directed to the corresponding author and Hearing Australia Human Research Ethics Committee.

## Abbreviations

The following abbreviations are used in this manuscript:

4FA HL	Four frequency averaged hearing loss at 0.5, 1, 2, and 4 kHz
AI	Artificial intelligence
ASR	Automatic speech recognition
BIT	Beginners Intelligibility Test
CIs	Cochlear implants
DNN	Deep neural network
HAs	Hearing aids
HL	Hearing loss
ICC	Intraclass correlation coefficient
LoA	Limits of agreement
NAL	National Acoustic Laboratories
NLP	Natural language processing
NH	Normal hearing
PTA	Pure tone audiometry
RMS	Root-mean-square
RNN	Recurrent neural network
SI	Speech intelligibility
SOTA	State-of-the-art
STT	Speech-to-text
WER	Word Error Rate
